# Nano-vehicles give new lease of life to existing antimicrobials

**DOI:** 10.1042/ETLS20200153

**Published:** 2020-12-01

**Authors:** Ioanna Mela, Clemens F. Kaminski

**Affiliations:** Department of Chemical Engineering and Biotechnology, University of Cambridge, Philippa Fawcett Drive, Cambridge CB3 0AS, U.K.

**Keywords:** antibiotic resistance, antibiotics, antimicrobial peptides, nano, nanotechnology, synthetic DNA

## Abstract

Antibiotic resistance has become one of the greatest challenges for modern medicine, and new approaches for the treatment of bacterial infections are urgently needed to avoid widespread vulnerability again to infections that have so far been easily treatable with existing drugs. Among the many approaches investigated to overcome this challenge is the use of engineered nanostructures for the precise and targeted delivery of existing antimicrobial agents in a fashion that will potentiate their effect. This idea leans on lessons learned from pioneering research in cancer, where the targeted delivery of anti-cancer drugs to mammalian cells has been a topic for some time. In particular, new research has demonstrated that nanomaterials can be functionalised with active antimicrobials and, in some cases, with targeting molecules that potentiate the efficiency of the antimicrobials. In this mini-review, we summarise results that demonstrate the potential for nanoparticles, dendrimers and DNA nanostructures for use in antimicrobial delivery. We consider material aspects of the delivery vehicles and ways in which they can be functionalised with antibiotics and antimicrobial peptides, and we review evidence for their efficacy to kill bacteria both *in vitro* and *in vivo*. We also discuss the advantages and limitations of these materials and highlight the benefits of DNA nanostructures specifically for their versatile potential in the present context.

## Introduction

Antibiotic resistance is a worldwide and growing human health issue, with major socio-economic impact. Significant work is being done to find ways to overcome this threat, but no definitive solution to this pressing healthcare challenge has yet been found. One promising line of research is the delivery of antimicrobials with nanomaterials. In particular, the evidence is accumulating that delivery of antimicrobials via delivery vehicles with ‘nanopatterned’ active molecules on their surface can potentiate the effects of the antimicrobials and increase their efficiency against microbial targets [1,2].

Nanoengineering enables the positioning of drugs at multiple sites on molecular scaffolds, which can be functionalised for targeted delivery of their payload. Delivery of such multivalent anti-cancer drugs has been explored in mammalian cells in several studies [3–7], but little work has been done in the field of multivalent antimicrobial delivery. With this mini-review, we illustrate how the delivery of active antimicrobials on the surface of nanostructures can potentiate the effects of existing antimicrobials.

**Figure 1.**
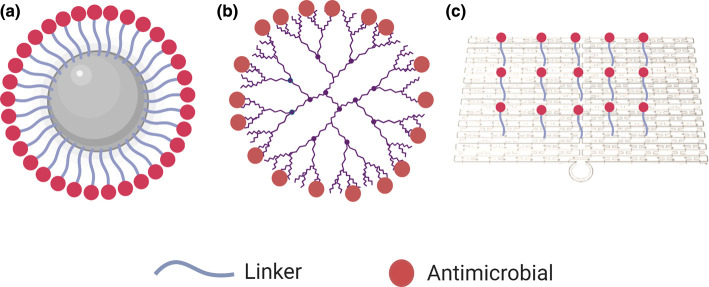
Nanomaterials that have potential for surface modification and drug delivery. (a) Nanoparticles, (b) dendrimers and (c) DNA nanostructures.

We first briefly consider the types of antimicrobials that have been used for this purpose, before discussing a range of materials that have been ‘nanopatterned’ with antimicrobials, including nanoparticles, dendrimers and DNA nanostructures (Figure 1), the degree of specificity and precision with which the antimicrobials can be patterned on the surface of these vehicles, and how these approaches potentiate the effects of the antimicrobials. We also comment on the most promising approaches and future directions for this field. We do not consider materials that encapsulate drugs for delivery, such as liposomes and micelles, but focus on materials that have the potential for surface modification with antimicrobials with varying degrees of precision.

### Antimicrobials used for ‘nanopatterned’ delivery

Two main categories of antimicrobials have been combined with nanomaterials to enable the delivery of multiple copies: antibiotics and antimicrobial peptides (AMPs). Antibiotics are generally classed according to their mechanism of action against bacteria. These broad categories include cell wall synthesis inhibitors, protein synthesis inhibitors, and DNA or RNA synthesis inhibitors. Antibiotics are small molecules that can be covalently linked or adsorbed onto a variety of nanomaterials [8,9].

AMPs, also known as host defence peptides, are produced by all life forms [10] and protect against infection. Each peptide has a broad range of antimicrobial activity usually against both Gram-negative and Gram-positive bacteria [11]. The key to the antimicrobial activity of AMPs is their interaction with the bacterial membrane. AMPs cause disruption to the microbial membrane and can cause bacterial cell death through lysis, or they can translocate into the cytoplasm and attack intracellular targets [12,13]. AMPs have low metabolic stability due to enzymatic degradation [14], which limits their use as antimicrobials. However, the use of nano-vehicles for their delivery can overcome this issue and potentiate the activity of AMPs. This potentiation is attributed to increased stability of the complexes against protease degradation in the matrix and/or inside the cells and against peptide self-aggregation [15]. It has been suggested that the high surface coverage and high charge density of the peptides when attached on nanoparticles is what protects the peptides against digestion by enzymes [16].

In the sections below, we explore the materials that have been explored for use as nano-vehicles for the delivery of antibiotics and AMPs, thereby potentiating the effects of these antimicrobials.

## Nanoparticles

Nanoparticles — defined as particles that range between 1 and 100 nm in diameter — are currently the most widely used vehicles for the delivery of active antimicrobials attached to their surface. Several properties of nanoparticles make them attractive candidates for antimicrobial delivery, including their high surface-to-volume ratio, the possibility of surface functionalisation, and their net-positive charge that allows them to bind to the negatively charged membrane of bacteria [17,18]. Many nanoparticles, especially metallic ones, possess antimicrobial properties themselves, however, for the purpose of this review we will focus on their potential as antimicrobial delivery vehicles. In this section, we will focus on metallic, silica-based or polymer-based nanoparticles [2,17,19].

Metallic nanoparticles are usually synthesised via chemical reduction in an inorganic salt (gold, silver, aluminium, titanium and others) and have the highest drug-loading efficiency of all types of nanoparticles [20]. Silica nanoparticles are most widely produced using the sol-gel method [21]. Polymer-based nanoparticles are composed of a polymeric matrix and can be prepared via different methods, such as solvent evaporation or diffusion and polymerisation. Some of the most commonly used polymers are polylactide (PLA), poly(lactic-co-glycolic acid) (PLGA) or natural ones, such as alginate and chitosan [22,23]. Antimicrobial drugs can be attached to polymeric nanoparticles through covalent bonding, hydrophobic interactions or entrapment [24].

**Figure 2.**
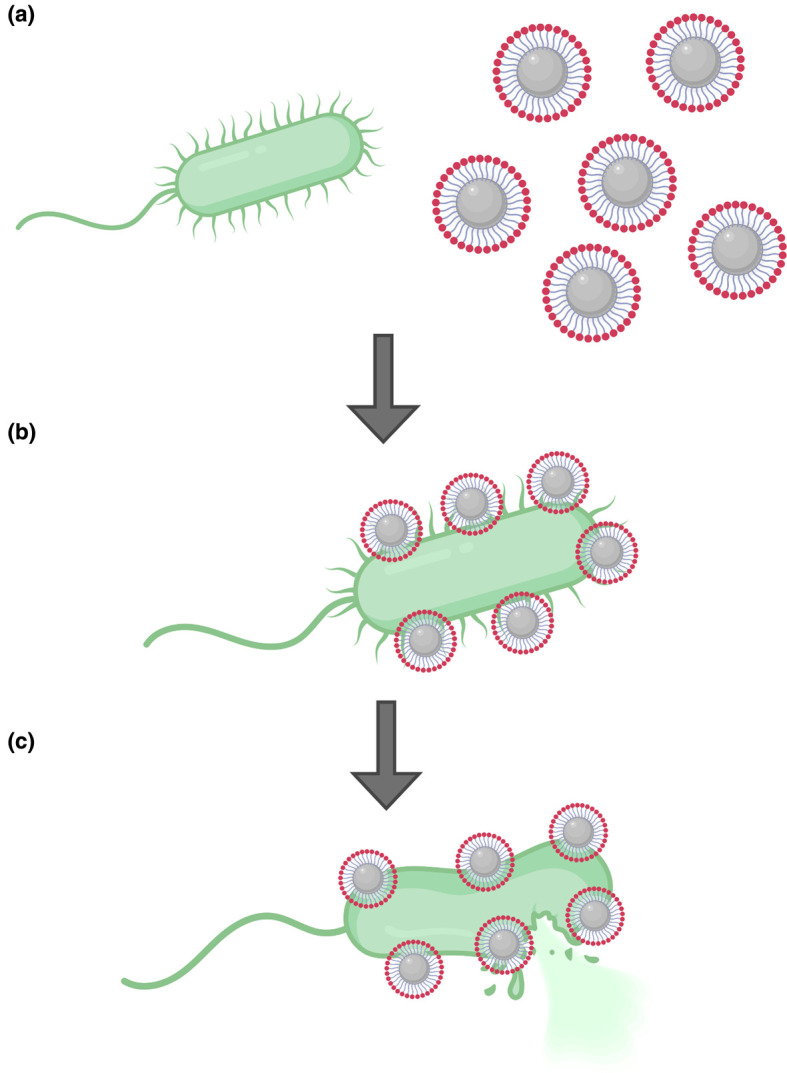
Nanoparticles as antimicrobial delivery vehicles. (**a**) Nanoparticles coated with antimicrobials can bind bacterial targets. (**b**) The antimicrobial cargo acts on the bacterial surface, sometimes synergistically with the nanoparticle carrier. (**c**) The synergistic effects between the antimicrobials and the nanoparticles and the potentiation of the active antimicrobial lead to bacterial cell lysis.

Several studies have demonstrated that the delivery of antibiotics and AMPs on the surface of nanoparticles increases their efficiency relative to that of the free antimicrobials (Figure 2). The mechanism of action of the nanoparticle–antimicrobial conjugates largely depends on the antimicrobial used in each case, but the potentiation of the activity of the antimicrobial substances is generally attributed to the protection of the active antimicrobial and synergistic effects between the active antimicrobial and the nanoparticle [15].

### Gold nanoparticles

Several studies have shown that the effects of antibacterial peptides can be potentiated *in vitro* and *in vivo* by their attachment to gold nanoparticles. Chen et al. attached surfactin, a cyclic peptide that penetrates cellular membranes, to ultrasmall Au nanodots via nonspecific hydrophobic interactions. Attachment of the peptide to the nanodots increased its efficiency against non-multidrug-resistant (*Escherichia coli*, *Proteus vulgaris*, *Salmonella enteritidis* and *Staphylococcus aureus*) and multidrug-resistant bacteria, such as MRSA, by 80-fold in comparison with its delivery free in solution. The increased antimicrobial effect was attributed to the synergistic effect of the peptide and the Au nanodots on the disruption of the bacterial membrane. Interestingly, a study in rats showed that the same system could promote healing of MRSA-infected wounds *in vivo* [25]. Similar *in vitro* and *in vivo* effects were observed for the AMP cecropin-melittin when delivered on larger Au nanoparticles of 14 nm*.* The effects of the peptide against *E. coli*, *Klebsiella pneumoniae*, *Pseudomonas aeruginosa* and *S. aureus* bacteria were potentiated *in vitro* and this antimicrobial activity was retained *in vivo* when used in wounds infected by *P. aeruginosa* and MRSA in mice [26]. The activity of esculentin-1a, a peptide derived from frog skin, against planktonic and biofilm-forming *P. euruginosa* was increased by 15-fold when covalently linked to Au nanoparticles*.* The peptide–nanoparticle conjugate was able to disrupt the bacterial membrane and cause lysis of bacterial cells [27]. Similarly, the activity of the AMP indolicidin [28] against *Candida albicanis* biofilms was potentiated when conjugated on Au nanoparticles.

Studies have also shown that the effects of existing antibiotics can be potentiated when delivered on nanoparticles. Work on antibiotics that are covalently bound on Au nanoparticles has been reviewed in detail by Vigderman and Zubarev [8]. Briefly, conjugation with nanoparticles has been shown to potentiate the effects of vancomycin, ampicillin, streptomycin and kanamycin against Gram-negative and Gram-positive pathogenic bacteria [8]. In 2002, Lin et al. produced gold nanoparticles that had been patterned with mannose, which binds specifically to FimH adhesin, a protein found on *E. coli* type 1 pili. The mannose–Au nanoparticles bound to this target selectively and significantly more efficiently than free mannose [29]. Phosphatidylcholine-decorated Au nanoparticles loaded with the antibiotic gentamicin are active against Gram-negative and Gram-positive bacteria, and are especially efficient in the prevention and disruption of biofilms [30].

A substantial body of work has also emerged on Au nanoclusters and has been recently reviewed by Yougbare et al. [31]*.* Au nanoclusters are ultra-small Au nanoparticles that consist of tens to hundreds of gold atoms. These nanoclusters have significant antimicrobial activity even when not functionalised with active antimicrobials, due to electrostatic interactions with the bacterial cell membrane [32]. However, conjugation of antimicrobial small molecules or macromolecules to Au nanoclusters can increase the potency of the multivalent, nanopatterned antimicrobials in comparison with that of the free active components [31].

### Silver nanoparticles

Silver (Ag) nanoparticles have also been used as antimicrobials, mostly exploiting the antimicrobial properties of silver itself [33,34], but also as vehicles for AMPs and antibiotics attached to their surface. As with Au nanoparticles, conjugation of AMPs to Ag nanoparticles increases the efficiency of these peptides against pathogenic bacterial strains [35]. For example, the potency of the cationic peptide odorranain-A-OA1 against *E. coli in vitro* was increased when the peptide was conjugated to Ag nanoparticles [36]. Ag nanoparticles have also been used as delivery platforms for photosensitising molecules: Ag nanoparticles conjugated with porphyrin have been shown to be bactericidal against *Staphylococcus epidermidis* and *E. coli* upon light activation [37].

Similar studies have been done with conjugates of Ag nanoparticles with other antimicrobials. In a study from Mei et al., Ag nanoparticles were combined with the antimicrobial chitosan to produce a synergistic antibacterial effect. The functionalised, multivalent nanoparticles bound to Mg^2+^ ions on the bacterial membrane and induced damage [38]. In another study, Ag nanoparticles capped with hydrolysed collagen and blends of collagen with other natural polymers were shown to have inhibitory activity against *E. coli* and *P. aeruginosa*, and these nanoparticles had low cytotoxicity against murine macrophages [39]. In a study by Khatoon et al. [40], the efficiency of ampicillin–Ag nanoparticles against drug-resistant bacteria was higher than that of the free antibiotic, and the toxicity of these nanoparticles to mammalian cells was minimal . Similarly, the efficiency of the antibiotic cefotaxime against strains of *E. coli* and MRSA was also increased when it was conjugated to Ag nanoparticles [41], and a synergistic antibacterial effect has been reported with lysozyme-Ag nanoparticle conjugates —the presence of these conjugates significantly decreased bacterial cell growth [42]. In one study, Ag nanoparticles were coated with mesoporous silica that was functionalized with ampicillin; these nanoparticles had good antibacterial properties and are a step towards overcoming some limitations of metallic nanoparticles, such as low colloidal stability and aggregation in biological environments [43].

### Other metallic nanoparticles

Though Au and Ag nanoparticles are the most commonly used as antimicrobial vehicles, other metallic nanoparticles have also been used. *In vitro* experiments have shown that Zinc oxide nanoparticles combined with polymerised geranium oil, which is antibacterial, have good antimicrobial performance against *E. coli* and *S. aureus* [44]. The same two bacteria have been found to be susceptible to Cu nanoparticles chelated to the antimicrobial catechin; the chelation protected catechin from oxidation and increased its bactericidal activity *in vitro* [45]. Finally, AMPs have been used in combination with alumina nanoparticles. The peptide EAAA-BP100 was covalently bound to the surface of the nanoparticles, and the antimicrobial activity of the bound peptide against *E. coli* and *Salmonella typhimurium* was greater than that of the free peptide [15].

### Silica nanoparticles

Silica nanoparticles have most commonly been used as mesoporous particles into which antimicrobial components can be loaded [46], but some studies have been done in which antimicrobials have been attached to their surface. An interesting study by Syryamina et al. showed that the antimicrobials trichogin GA IV and ampullosporin A can be adsorbed on the surface of colloidal silica. The peptides formed close-packed clusters on the surface of the nanoparticles, although these functionalised nanoparticles were not tested against bacteria [47]. Previously, a study by Li and Wang showed that lysozyme-coated silica nanoparticles are effective against *E. coli in vitro* and in an intestine-infected mouse model with minimal side effects [48]. In another study, vancomycin-modified silica nanoparticles were used to specifically target and kill Gram-positive bacteria [40]. This specificity of vancomycin for Gram-positive bacteria over Gram-negative bacteria and other cells is due to the specific hydrogen-bonding interactions of vancomycin toward the terminal d-alanyl-d-alanine moieties of Gram-positive bacteria [49]. Multivalent vancomycin is active against vancomycin-resistant bacterial strains [50], and vancomycin-modified silica particles have the potential to overcome this vancomycin resistance by delivering multiple vancomycin molecules to the bacterial target. In a study published in 2020, this use of vancomycin-loaded silicon nanoparticles was refined by the addition of the peptide CARG to the surface to facilitate bacterial targeting. CARG can recognise *S. aureus* bacteria and facilitate the accumulation of systemically administered payloads (ranging from drug-sized molecules to nanoparticles) at sites of staphylococcal infection [51]. The silicon nanoparticles loaded with vancomycin and targeted with the CARG peptide were significantly more effective than free vancomycin at eradicating a staphylococcal lung infection in mice.

### Polymeric nanoparticles

Polymer-based nanoparticles have also attracted interest in the context of antimicrobial delivery. For example, polyion complex (PIC) nanoparticles carrying the last resort antimicrobial polymyxin B (Pol-B) had a sustained inhibitory effect on the growth of *P*. *aeruginosa* [52]*.* Similarly, polymer nanoparticles decorated with different densities of poly(dimethylaminoethyl methacrylate) have antimicrobial activity against *E. coli* and *Mycobacterium smegmatis* [53]. Interestingly, the mechanism of action against the two bacterial strains differed — the nanoparticles were bactericidal against *E. coli* and bacteriostatic against *M. smegmatis*.

Delivery of multiple active molecules on polymeric nanoparticles has also been shown to be effective against biofilms. For example, the synthetic peptide BAR was more effective at disrupting *Porphyromonas gingivalis* and *Streptococcus gordonii* biofilms when delivered on PGLA nanoparticles than when free [54]. Interestingly, similar effects have been observed with the delivery of multiple copies of photosensitisers to bacterial targets. In a study by Staegemann et al., hyperbranched polyglycerol (hPG) loaded with zinc porphyrin photosensitisers and mannose was used against *S. aureus* targets. The conjugates had significant antibacterial activity when carrying 70 or more mannose units, showing that the targeted delivery of photosensitisers can also potentiate photodynamic therapy [55].

Finally, in an interesting study by Laroque, Reifarth et al., AMPs were used not in conjunction with nanoparticles and fibres, but in a ‘bottlebrush’ architecture, where polymeric side chains are attached to a backbone. This material interfered with the bacterial cell envelope and affected bacterial growth [56]. Similar effects were reported by Zhang et al. [57].

### Advantages and challenges

Overall, nanoparticles have a range of properties that make them promising antimicrobial vehicles, such as high surface area, stability and low production costs. The range of sizes of nanoparticles currently used in antimicrobial delivery applications is between 10 and 100 nm, with varying shapes. The range of sizes and shapes is indicative of the versatility of nanoparticles; however, no studies have been done to directly compare the effects of size and shape on efficacy. More work on this area could inform future studies, although the shape of nanoparticles cannot currently be controlled easily. Au, silica and polymeric nanoparticles generally have good biocompatibility and relatively low toxicity to mammalian cells [58]. However, the vast majority are non-biodegradable and their accumulation in organs can become toxic in the long term. Moreover, the exact position of the active molecules on the nanoparticles is difficult to control and this means that multiple types of active molecules cannot be easily or precisely attached to one nanoparticle. This makes the functionalisation of nanoparticles with combinations of active molecules — either ‘cocktails’ of antimicrobials or combinations of antimicrobial and targeting molecules — extremely challenging.

## Dendrimers and dendrimeric peptides

Another category of materials that have shown promise as delivery vehicles is that of dendrimers. Dendrimers are highly branched, globular, polymeric structures (Figure 1b) that are usually made of poly(amidoamine) (PAMAM), poly(propyleneimine) (PPI) or poly(ethylene glycol) (PEG). They have three distinct structural features: a central core; surface functionalities; and branching units that link the two. The branching units are identical fragments that build up from the core to make star-like macromolecular structures [59]. Dendrimers are normally nano-scale in size and they have narrow polydispersity, uniform nanomorphology and tunable surface entities. The terminal groups at the arms of the dendrimer determine its solubility, reactivity and potential for further modification [60]. Dendrimers themselves have shown potential as selective antibacterials that mimic the action of AMPs, associating with and disrupting bacterial membranes through cationic and hydrophobic interactions [61,62], but they can also potentiate the effects of antimicrobials. They have been used as encapsulation agents, but here we focus on approaches that enable the delivery of multiple antimicrobial molecules on the surface of the dendrimer [63,64].

### Dendrimer–antimicrobial conjugates

A variety of antibiotics and AMPs have been conjugated to dendrimers in attempts to potentiate their antimicrobial effects. Penicillin that was conjugated to a PAMAM dendrimer via a PEG linker retained its activity, and this system was effective against *S. aureus* when designed so that the penicillin stayed bound to the dendrimer and when designed so that penicillin was released in close proximity to the bacteria [65]. Choi et al. developed dendrimer-based vancomycin conjugates against Gram-positive bacteria, and these nanostructures bound to vancomycin-resistant and non-resistant *S. aureus* with nanomolar affinity [49]. In a recent study, the antibiotic ciprofloxacin was conjugated to a G0-PAMAM dendrimer, and the antimicrobial activity of the conjugate was greater than that of free ciprofloxacin against *Enterococcus faecalis*, *S. aureus* and *P. aeruginosa* [66]. Glycodendrimers and phosphorus dendrimers have also been used in combination with the antibiotic levofloxacin. The synergistic action of the dendrimers and the antibiotic significantly increased antibacterial efficiency against *E. coli* in comparison with the free antibiotic [67]*.* AMPs have also been combined with dendrimers to potentiate their effects — peptides that were covalently conjugated with cationic carbosilane dendrons caused significant damage to bacterial membranes in *E. coli* and *S. aureus in vitro* [68]. Finally, Kitov et al. have developed a lactose-based polymeric nanosystem that carries multivalent, templated inhibitors of Shiga toxin (which is produced by *E. coli*) [69]. This system increased the activity of the inhibitors by several orders of magnitude *in vivo*.

### Advantages and challenges

Dendrimers offer a range of advantages as nano-vehicles, the main one being precise control of the size of the dendrimer and the number of active molecules that can be attached on its surface. They can also increase the solubility of antimicrobials and, due to their modular nature, their chemical and physical properties are tunable. However, dendrimers are expensive to produce, and can be cytotoxic due to interactions between the positively charged dendrimers and the negatively charged cell membrane [70].

## DNA nanostructures

**Figure 3.**
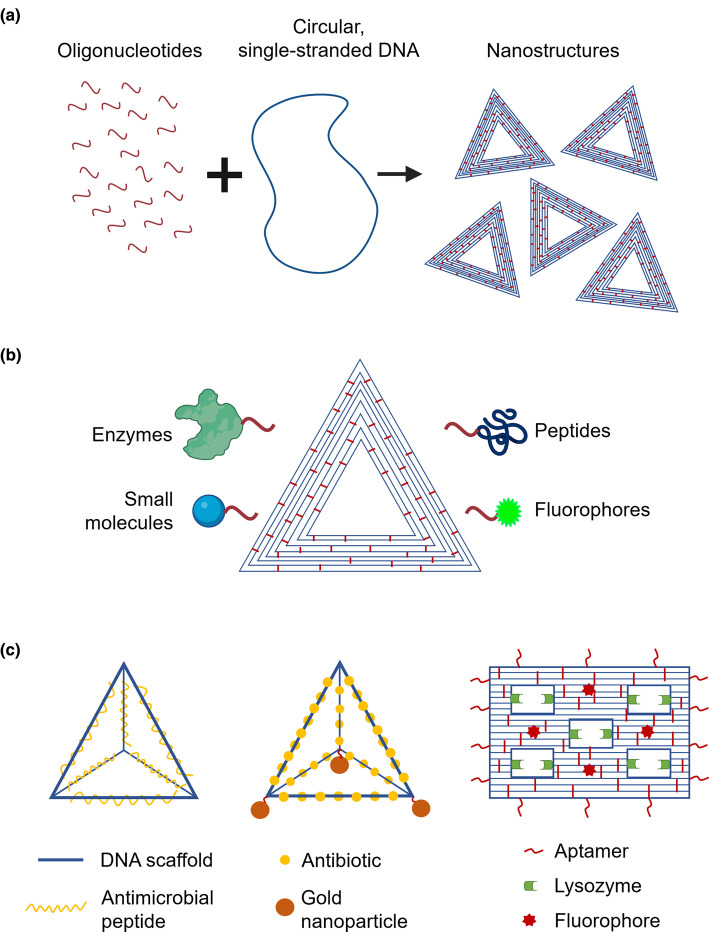
DNA nanostructures have potential as antimicrobial delivery vehicles. (**a**) Hundreds of oligonucleotides (red) bind to pre-designed positions of a long, circular, single-stranded piece of DNA (blue), folding it and holding it in place to form a nanostructure. (**b**) Each one of the oligonucleotides (red) can be functionalised so that it carries active molecules, such as antimicrobials (enzymes, small molecules, peptides) or fluorophores. (**c**) DNA nanostructures that have been used to date as antimicrobial carriers. DNA pyramids have been combined with antimicrobial peptides (left) or the antibiotic actinomycin and gold nanoparticles (centre), while a ‘five-well frame’ nanostructure (right) has been used to deliver the antimicrobial enzyme lysozyme and at the same time specifically bind bacterial targets using aptamers.

An emerging category of antimicrobial delivery vehicle is DNA nanostructures. These nanostructures are made by folding a long piece of single-stranded, circular DNA into pre-designed shapes, first proposed by Seeman in the 1980s [71,72] and revolutionised by Rothemund in 2006 [73]. The structures are held together by hundreds of oligonucleotides (Figure 3a) that can be functionalised to carry a wide variety of active molecules (Figure 3b). Each oligonucleotide can be functionalised independently, so that the nanostructures can simultaneously carry multiple different molecules that are precisely positioned on the structure's surface [74–78]. Another advantage of DNA nanostructures is their excellent biocompatibility; though they can have low stability in biological fluids, mostly due to susceptibility to DNA-targeting enzymes present, this stability can be improved with chemical modifications [79].

A few studies have clearly demonstrated the potential of DNA nanostructures as antimicrobial delivery vehicles. In 2014, Setyawti and colleagues showed that tetrahedral DNA nanostructures loaded with Au nanoclusters as detection beacons and with actinomycin as the active antimicrobial (Figure 3c) can be taken up by *E. coli* and *S. aureus*. Treatment with the nanostructure reduced the bacterial population by 65%, whereas the free antibiotic reduced the population by 42%. The increased activity of the bound drug was attributed to its localised release from the DNA vehicle via degradation of the DNA by DNAses present in the bacteria [80]. More recently, tetrahedral DNA nanostructures have been used by Liu et al. to deliver the AMP GL13K (Figure 3c) to *E. coli* and *P. gingivalis.* The nanostructures increased bacterial uptake of the peptide, increased the stability of the peptide by protecting against proteases in the extracellular environment, and potentiated the effects of the peptide against both bacterial targets [81].

DNA origami has recently been used as a delivery vehicle for the antimicrobial enzyme lysozyme. In this study, Mela et al. exploited the potential for simultaneous multi-functionalisation of DNA origami by adding three functional components: aptamers that specifically bind to *E. coli* and *Bacillus subtilis*, the antimicrobial enzyme, and fluorophores to act as detection beacons for the nanostructures (Figure 3c). The nanostructures specifically bound to their bacterial targets, and the origami-bound lysozyme was more efficient at slowing bacterial growth than free lysozyme [82].

These studies show that the ability to relatively easily modify and functionalise DNA nanostructures makes them promising nano-vehicles that enable controlled, precise and targeted delivery of antimicrobials. Any desired functionality can be added to the DNA nanostructures by attaching enzymes, proteins, drugs, peptides and other functional molecules to them with nanometre precision. This precision allows for control of the spatial relationships between each of the functional molecules and between the functional molecules and their targets, thereby providing fine control over their interactions. Consequently, DNA nanostructures have enormous potential for tuneable microbial targeting applications and could hold the key to overcoming the theranostic challenges posed by growing antibiotic resistance.

## Conclusion

Antibiotic resistance remains a world-wide health issue that is rendering us once again vulnerable to diseases that have been treatable for decades. A wealth of research is emerging from efforts to overcome this threat, including attempts to deliver multiple copies of one or more antimicrobials in a patterned, precise, and targeted manner. Nanomaterials are showing great potential as vehicles for the precise delivery of active antimicrobials and the potentiation of their effects against their targets. Despite disadvantages such as potential cytotoxicity, in the case of nanoparticles and dendrimers and limited stability in biological fluids in the case of DNA nanostructures, the concept of delivering antimicrobials through nanostructures is gaining momentum. Further research is now needed to understand how to overcome the limitations of different nanocarriers and how exactly the targeted delivery of antimicrobials potentiates their effects so that these effects can be maximised. Also, characterisation of the pharmacokinetic and pharmacodynamic properties of antibacterial nanocarriers need to be studied for those materials to be successfully translated into successful candidates in *in vivo* drug delivery.

Developments in DNA nanotechnology have enabled the delivery of antimicrobials with nano-vehicles, which is becoming increasingly precise. This fine-tuning of the delivery provides more and more control over which antimicrobials are delivered, in what combinations and in what numbers to their bacterial targets. DNA nanostructures show particular promise due to their biocompatibility and high functionalisation potential. They provide a platform that is highly versatile and can be functionalised to simultaneously carry antimicrobials, photosensitisers, detection beacons such as fluorophores, and aptamers and antibodies that allow for specific sensing and binding of bacterial targets. The future of antibiotics likely lies in multivalent, targeted and precise delivery and more research is needed in this field to understand and gain full control over the ways in which DNA nanostructures can become potent antibacterial nano-vehicles.

## Summary

- Antibiotic resistance is one of the greatest challenges in modern medicine. New approaches to the treatment of bacterial infections are urgently needed.
- Use of ‘nano-vehicles’ for precise, targeted delivery of antimicrobial agents to potentiate their effects shows promise.
- Nanoparticles, dendrimers and DNA nanostructures can be used for this type of antimicrobial delivery.
- Of those, DNA nanostructures show exceptional potential for functionalisation with active antimicrobials and targeting molecules to increase the efficiency of the antimicrobials.
- Ongoing studies are exploring the development and optimisation of DNA nanostructures as antimicrobial delivery vehicles.

## Abbreviations

AMPs: antimicrobial peptides
PAMAM: poly(amidoamine)
PEG: poly(ethylene glycol)

## References

[1] Kumar M., Curtis A. and Hoskins C. (2018) Application of nanoparticle technologies in the combat against anti-microbial resistance. Pharmaceutics 10, 11 10.3390/pharmaceutics10010011 PMC5874824 29342903

[2] Martin-Serrano Á., Gómez R., Ortega P. and La M.F. (2019) Nanosystems as vehicles for the delivery of antimicrobial peptides (Amps). Pharmaceutics 11, 448 10.3390/pharmaceutics11090448 PMC6781550 31480680

[3] Csizmar C.M., Petersburg J.R., Perry T.J., Rozumalski L., Hackel B.J. and Wagner C.R. (2019) Multivalent ligand binding to cell membrane antigens: defining the interplay of affinity, valency, and expression density. J. Am. Chem. Soc. 141, 251–261 10.1021/jacs.8b09198 30507196 PMC6520051

[4] Zhang Q., Jiang Q., Li N., Dai L., Liu Q., Song L. et al. (2014) DNA origami as an *in vivo* drug delivery vehicle for cancer therapy. ACS Nano 8, 6633–6643 10.1021/nn502058j 24963790

[5] Li S., Jiang Q., Liu S., Zhang Y., Tian Y., Song C. et al. (2018) A DNA nanorobot functions as a cancer therapeutic in response to a molecular trigger in vivo. Nat. Biotechnol. 36, 258–264 10.1038/nbt.4071 29431737

[6] Guido C., Maiorano G., Cortese B., D'Amone S. and Palamà I.E. (2020) Biomimetic nanocarriers for cancer target therapy. Bioengineering 7, 111 10.3390/bioengineering7030111 PMC7552783 32937963

[7] Tian Y. and Zhou S. (2020) Advances in cell penetrating peptides and their functionalization of polymeric nanoplatforms for drug delivery. Wiley Interdiscip. Rev. Nanomed. Nanobiotechnol. e1668 32929866 10.1002/wnan.1668

[8] Vigderman L. and Zubarev E.R. (2013) Therapeutic platforms based on gold nanoparticles and their covalent conjugates with drug molecules. Adv. Drug. Deliv. Rev. 65, 663–676 10.1016/j.addr.2012.05.004 22613038

[9] Ji L., Liu F., Xu Z., Zheng S. and Zhu D. (2010) Adsorption of pharmaceutical antibiotics on template-synthesized ordered micro- and mesoporous carbons. Environ. Sci. Technol. 44, 3116–3122 10.1021/es903716s 20201519

[10] Hancock R.E.W. (2000) Cationic antimicrobial peptides: towards clinical applications. Expert Opin. Investig. Drugs 9, 1723–1729 10.1517/13543784.9.8.1723 11060771

[11] Marr A.K., Gooderham W.J. and Hancock R.E. (2006) Antibacterial peptides for therapeutic use: obstacles and realistic outlook. Curr. Opin. Pharmacol. 6, 468–472 10.1016/j.coph.2006.04.006 16890021

[12] Malmsten M. (2015) Interactions of antimicrobial peptides with bacterial membranes and membrane components. Curr. Top. Med. Chem. 16, 16–24 10.2174/1568026615666150703121518 26139113

[13] Yeung A.T.Y., Gellatly S.L. and Hancock R.E.W. (2011) Multifunctional cationic host defence peptides and their clinical applications. Cell. Mol. Life Sci. 68, 2161–2176 10.1007/s00018-011-0710-x 21573784 PMC11114888

[14] Vlieghe P., Lisowski V., Martinez J. and Khrestchatisky M. (2010) Synthetic therapeutic peptides: science and market. Drug Discov. Today 15, 40–56 10.1016/j.drudis.2009.10.009 19879957

[15] Torres L.M.F.C., Braga N.A., Gomes I.P., Almeida M.T., Santos T.L., de Mesquita J.P. et al. (2018) Nanobiostructure of fibrous-like alumina functionalized with an analog of the BP100 peptide: synthesis, characterization and biological applications. Colloids Surf. B Biointerfaces 163, 275–283 10.1016/j.colsurfb.2018.01.001 29329073

[16] Wadhwani P., Heidenreich N., Podeyn B., Bürck J. and Ulrich A.S. (2017) Antibiotic gold: tethering of antimicrobial peptides to gold nanoparticles maintains conformational flexibility of peptides and improves trypsin susceptibility. Biomater. Sci. 5, 817–827 10.1039/C7BM00069C 28275774

[17] Yeh Y.C., Huang T.H., Yang S.C., Chen C.C. and Fang J.Y. (2020) Nano-based drug delivery or targeting to eradicate bacteria for infection mitigation: a review of recent advances. Front. Chem. 8, 286 10.3389/fchem.2020.00286 32391321 PMC7193053

[18] Zazo H., Colino C.I. and Lanao J.M. (2016) Current applications of nanoparticles in infectious diseases. J. Control. Release 224, 86–102 10.1016/j.jconrel.2016.01.008 26772877

[19] Gao W., Chen Y., Zhang Y., Zhang Q. and Zhang L. (2018) Nanoparticle-based local antimicrobial drug delivery. Adv. Drug Deliv. Rev. 127, 46–57 10.1016/j.addr.2017.09.015 28939377 PMC5860926

[20] Turkevich, J. (1985) Colloidal Gold. Part II Colour, Coagulation, Adhesion, Alloying And Catalytic Properties, pp. 125–131, vol. 18, Springer 10.1007/BF03214694

[21] Rahman I.A. and Padavettan V. ( 2012) Synthesis of silica nanoparticles by sol-gel: size-dependent properties, surface modification, and applications in silica-polymer nanocompositesa review. J. Nanomater. 2012, 132424 10.1155/2012/132424

[22] Gonçalves I.C., Henriques P.C., Seabra C.L. and Martins M.C.L. (2014) The potential utility of chitosan micro/nanoparticles in the treatment of gastric infection. Expert Rev. Anti-Infect. Ther. 12, 981–992 10.1586/14787210.2014.930663 24981812

[23] Lembo D. and Cavalli R. (2010) Review nanoparticulate delivery systems for antiviral drugs. Antivir. Chem. Chemother. 21, 53–70 10.3851/IMP1684 21107015

[24] Bamrungsap S., Zhao Z., Chen T., Wang L., Li C., Fu T. et al. (2012) Nanotechnology in therapeutics: a focus on nanoparticles as a drug delivery system. Nanomedicine 7, 1253–1271 10.2217/nnm.12.87 22931450

[25] Chen W.Y., Chang H.Y., Lu J.K., Huang Y.C., Harroun S.G., Tseng Y.T. et al. (2015) Self-assembly of antimicrobial peptides on gold nanodots: against multidrug-resistant bacteria and wound-healing application. Adv. Funct. Mater. 25, 7189–7199 10.1002/adfm.201503248

[26] Rai A., Pinto S., Velho T.R., Ferreira A.F., Moita C., Trivedi U. et al. (2016) One-step synthesis of high-density peptide-conjugated gold nanoparticles with antimicrobial efficacy in a systemic infection model. Biomaterials 85, 99–110 10.1016/j.biomaterials.2016.01.051 26866877

[27] Casciaro B., Moros M., Rivera-Fernández S., Bellelli A., de la Fuente J.M. and Mangoni M.L. (2017) Gold-nanoparticles coated with the antimicrobial peptide esculentin-1a(1-21)NH2 as a reliable strategy for antipseudomonal drugs. Acta Biomater. 47, 170–181 10.1016/j.actbio.2016.09.041 27693686

[28] de Alteriis E., Maselli V., Falanga A., Galdiero S., Di Lella F.M., Gesuele R. et al. (2018) Efficiency of gold nanoparticles coated with the antimicrobial peptide indolicidin against biofilm formation and development of Candida spp. clinical isolates. Infect Drug Resist. 11, 915–925 10.2147/IDR.S164262 30013374 PMC6037145

[29] Lin C.-C., Yeh Y.-C., Yang C.-Y., Chen C.-L., Chen G.-F., Chen C.-C. et al. (2002) Downloaded via UNIV OF CAMBRIDGE on. J. Am. Chem. Soc. 124, 24 10.1021/ja0171097 11772055

[30] Mu H., Tang J., Liu Q., Sun C., Wang T. and Duan J. (2016) Potent antibacterial nanoparticles against biofilm and intracellular bacteria. Sci. Rep. 6, 18877 10.1038/srep18877 26728712 PMC4700437

[31] Yougbare S., Chang T.K., Tan S.H., Kuo J.C., Hsu P.H., Su C.Y. et al. (2019) Antimicrobial gold nanoclusters: recent developments and future perspectives. Int. J. Mol. Sci. 20, 2924 10.3390/ijms20122924 PMC6627976 31208013

[32] Zhang S.S., Wang X., Su H.F., Feng L., Wang Z., Ding W.Q. et al. (2017) A water-stable Cl@Ag 14 cluster based metal-organic open framework for dichromate trapping and bacterial inhibition. Inorg. Chem. 56, 11891–11899 10.1021/acs.inorgchem.7b01879 28933555

[33] Prabhu S. and Poulose E.K. (2012) Silver nanoparticles: mechanism of antimicrobial action, synthesis, medical applications, and toxicity effects. Int. Nano Lett. 2, 32 10.1186/2228-5326-2-32

[34] Rai M., Yadav A. and Gade A. (2009) Silver nanoparticles as a new generation of antimicrobials. Biotechnol. Adv. 27, 76–83 10.1016/j.biotechadv.2008.09.002 18854209

[35] Pal I., Bhattacharyya D., Kar R.K., Zarena D., Bhunia A. and Atreya H.S. (2019) A peptide-nanoparticle system with improved efficacy against multidrug resistant bacteria. Sci. Rep. 9, 4485 10.1038/s41598-019-41005-7 30872680 PMC6418133

[36] Pal I., Brahmkhatri V.P., Bera S., Bhattacharyya D., Quirishi Y., Bhunia A. et al. (2016) Enhanced stability and activity of an antimicrobial peptide in conjugation with silver nanoparticle. J. Colloid Interface Sci. 483, 385–393 10.1016/j.jcis.2016.08.043 27585423

[37] Elashnikov R., Radocha M., Panov I., Rimpelova S., Ulbrich P., Michalcova A. et al. (2019) Porphyrin-silver nanoparticles hybrids: synthesis, characterization and antibacterial activity. Mater. Sci. Eng. C 102, 192–199 10.1016/j.msec.2019.04.029 31146990

[38] Mei L., Xu Z., Shi Y., Lin C., Jiao S., Zhang L. et al. (2020) Multivalent and synergistic chitosan oligosaccharide-Ag nanocomposites for therapy of bacterial infection. Sci. Rep. 10, 10011 10.1038/s41598-020-67139-7 32561796 PMC7305188

[39] Nogueira S.S., de Araujo-Nobre A.R., Mafud A.C., Guimarães M.A., Alves M.M.M., Plácido A. et al. (2019) Silver nanoparticle stabilized by hydrolyzed collagen and natural polymers: synthesis, characterization and antibacterial-antifungal evaluation. Int. J. Biol. Macromol. 135, 808–814 10.1016/j.ijbiomac.2019.05.214 31158421

[40] Khatoon N., Alam H., Khan A., Raza K. and Sardar M. (2019) Ampicillin silver nanoformulations against multidrug resistant bacteria. Sci. Rep. 9, 6848 10.1038/s41598-019-43309-0 31048721 PMC6497658

[41] Halawani E.M., Hassan A.M. and Gad El-Rab S.M. (2020) Nanoformulation of biogenic cefotaxime-conjugated-silver nanoparticles for enhanced antibacterial efficacy against multidrug-resistant bacteria and anticancer studies. Int. J. Nanomed. 15, 1889–1901 10.2147/IJN.S236182 PMC7090159 32256066

[42] Ernest V., Gajalakshmi S., Mukherjee A. and Chandrasekaran N. (2014) Enhanced activity of lysozyme-AgNP conjugate with synergic antibacterial effect without damaging the catalytic site of lysozyme. Artif Cells Nanomed. Biotechnol. 42, 336–343 10.3109/21691401.2013.818010 23863117

[43] de Oliveira J.F.A., Saito Â., Bido A.T., Kobarg J., Stassen H.K. and Cardoso M.B. (2017) Defeating bacterial resistance and preventing mammalian cells toxicity through rational design of antibiotic-functionalized nanoparticles. Sci. Rep. 7, 1326 10.1038/s41598-017-01209-1 28465530 PMC5430956

[44] Al-Jumaili A., Mulvey P., Kumar A., Prasad K., Bazaka K., Warner J. et al. (2019) Eco-friendly nanocomposites derived from geranium oil and zinc oxide in one step approach. Sci. Rep. 9, 5973 10.1038/s41598-019-42211-z 30979934 PMC6461640

[45] Li H., Chen Q., Zhao J. and Urmila K. (2015) Enhancing the antimicrobial activity of natural extraction using the synthetic ultrasmall metal nanoparticles. Sci. Rep. 5, 11033 10.1038/srep11033 26046938 PMC4457014

[46] Park S.Y., Barton M. and Pendleton P. (2011) Mesoporous silica as a natural antimicrobial carrier. Colloids Surfaces A Physicochem. Eng. Asp. 385, 256–261 10.1016/j.colsurfa.2011.06.021

[47] Syryamina V.N., Samoilova R.I., Tsvetkov Y.D., Ischenko A V., De Zotti M., Gobbo M. et al. (2016) Peptides on the surface: spin-label EPR and PELDOR study of adsorption of the antimicrobial peptides trichogin GA IV and ampullosporin a on the silica nanoparticles. Appl. Magn. Reson. 47, 309–320 10.1007/s00723-015-0745-5

[48] Li L. and Wang H. (2013) Enzyme-coated mesoporous silica nanoparticles as efficient antibacterial agents in vivo. Adv. Healthc. Mater. 2, 1351–1360 10.1002/adhm.201300051 23526816

[49] Choi S.K., Myc A., Silpe J.E., Sumit M., Wong P.T., McCarthy K. et al. (2013) Dendrimer-based multivalent vancomycin nanoplatform for targeting the drug-resistant bacterial surface. ACS Nano 7, 214–228 10.1021/nn3038995 23259666

[50] Silverman S.M., Moses J.E. and Sharpless K.B. (2017) Reengineering antibiotics to combat bacterial resistance: click chemistry [1,2,3]-triazole vancomycin dimers with potent activity against MRSA and VRE. Chemistry 23, 79–83 10.1002/chem.201604765 27747932

[51] Hussain S., Joo J., Kang J., Kim B., Braun G.B., She Z.-G. et al. (2018) Antibiotic-loaded nanoparticles targeted to the site of infection enhance antibacterial efficacy. Nat. Biomed. Eng. 2, 95–103 10.1038/s41551-017-0187-5 29955439 PMC6015743

[52] Insua I., Zizmare L., Peacock A.F.A., Krachler A.M. and Fernandez-Trillo F. (2017) Polymyxin B containing polyion complex (PIC) nanoparticles: improving the antimicrobial activity by tailoring the degree of polymerisation of the inert component. Sci. Rep. 7, 9396 10.1038/s41598-017-09667-3 28839223 PMC5570901

[53] Richards S.J., Isufi K., Wilkins L.E., Lipecki J., Fullam E. and Gibson M.I. (2018) Multivalent antimicrobial polymer nanoparticles target mycobacteria and gram-negative bacteria by distinct mechanisms. Biomacromolecules 19, 256–264 10.1021/acs.biomac.7b01561 29195272 PMC5761047

[54] Kalia P., Jain A., Radha Krishnan R., Demuth D.R. and Steinbach-Rankins J.M. (2017) Peptide-modified nanoparticles inhibit formation of *Porphyromonas gingivalis* biofilms with *Streptococcus gordonii*. Int. J. Nanomed. 12, 4553–4562 10.2147/IJN.S139178 PMC5488760 28790818

[55] Staegemann M.H., Gitter B., Dernedde J., Kuehne C., Haag R. and Wiehe A. (2017) Mannose-functionalized hyperbranched polyglycerol loaded with zinc porphyrin: investigation of the multivalency effect in antibacterial photodynamic therapy. Chemistry 23, 3918–3930 10.1002/chem.201605236 28029199

[56] Laroque S., Reifarth M., Sperling M., Kersting S., Klöpzig S., Budach P. et al. (2020) Impact of multivalence and self-assembly in the design of polymeric antimicrobial peptide mimics. ACS Appl. Mater Interfaces 12, 30052–30065 10.1021/acsami.0c05944 32517467

[57] Zhang Y., Kang K., Zhu N., Li G., Zhou X., Zhang A. et al. (2020) Bottlebrush-like highly efficient antibacterial coating constructed using α-helical peptide dendritic polymers on the poly(styrene-b-(ethylene-co-butylene)-b-styrene) surface. J. Mater. Chem. B 8, 7428–7437 32662494 10.1039/d0tb01336f

[58] Lewinski N., Colvin V. and Drezek R. (2008) Cytotoxicity of nanopartides. Small 4, 26–49 10.1002/smll.200700595 18165959

[59] Sadler K. and Tam J.P. (2002) Peptide dendrimers: applications and synthesis. Rev. Mol. Biotechnol. 90, 195–229 10.1016/S1389-0352(01)00061-7 12071226

[60] Walter M V. and Malkoch M. (2012) Simplifying the synthesis of dendrimers: accelerated approaches. Chem. Soc. Rev. 41, 4593–4609 10.1039/c2cs35062a 22592560

[61] Lopez A.I., Reins R.Y., McDermott A.M., Trautner B.W. and Cai C. (2009) Antibacterial activity and cytotoxicity of PEGylated poly(amidoamine) dendrimers. Mol. Biosyst. 5, 1148–1156 10.1039/b904746h 19756304 PMC2965593

[62] Schito A.M. and Alfei S. (2020) Antibacterial activity of non-cytotoxic, amino acid-modified polycationic dendrimers against *Pseudomonas aeruginosa* and other non-fermenting gram-negative bacteria. Polymers (Basel) 12, 1818 10.3390/polym12081818 PMC7464783 32823557

[63] Ma M., Cheng Y., Xu Z., Xu P., Qu H., Fang Y. et al. (2007) Evaluation of polyamidoamine (PAMAM) dendrimers as drug carriers of anti-bacterial drugs using sulfamethoxazole (SMZ) as a model drug. Eur. J. Med. Chem. 42, 93–98 10.1016/j.ejmech.2006.07.015 17095123

[64] Cheng Y., Qu H., Ma M., Xu Z., Xu P., Fang Y. et al. (2007) Polyamidoamine (PAMAM) dendrimers as biocompatible carriers of quinolone antimicrobials: an in vitro study. Eur. J. Med. Chem. 42, 1032–1038 10.1016/j.ejmech.2006.12.035 17336426

[65] Yang H. and Lopina S.T. (2003) Penicillin V-conjugated PEG-PAMAM star polymers. J. Biomater. Sci. Polym. Ed. 14, 1043–1056 10.1163/156856203769231556 14661878

[66] Svenningsen S.W., Frederiksen R.F., Counil C., Ficker M., Leisner J.J. and Christensen J.B. (2020) Synthesis and antimicrobial properties of a ciprofloxacin and PAMAM-dendrimer conjugate. Molecules 25, 1389 10.3390/molecules25061389 PMC7146445 32197523

[67] Wrońska N., Majoral J.P., Appelhans D., Bryszewska M. and Lisowska K. (2019) Synergistic effects of anionic/cationic dendrimers and levofloxacin on antibacterial activities. Molecules 24, 2894 10.3390/molecules24162894 PMC6719981 31395831

[68] Fernandez J., Acosta G., Pulido D., Malý M., Copa-Patiño J.L., Soliveri J. et al. (2019) Carbosilane dendron-peptide nanoconjugates as antimicrobial agents. Mol. Pharm. 16, 2661–2674 10.1021/acs.molpharmaceut.9b00222 31009225

[69] Kitov P.I., Mulvey G.L., Griener T.P., Lipinski T., Solomon D., Paszkiewicz E. et al. (2008) In vivo supramolecular templating enhances the activity of multivalent ligands: a potential therapeutic against the *Escherichia coli* O157 AB 5 toxins. Proc. Natl. Acad. Sci. U.S.A. 105, 16837–16842 10.1073/pnas.0804919105 18955695 PMC2573949

[70] Janaszewska A., Lazniewska J., Trzepiński P., Marcinkowska M. and Klajnert-Maculewicz B. (2019) Cytotoxicity of dendrimers. Biomolecules 9, 330 10.3390/biom9080330 PMC6723213 31374911

[71] Seeman N.C. (1982) Nucleic acid junctions and lattices. J. Theor. Biol. 99, 237–247 10.1016/0022-5193(82)90002-9 6188926

[72] Seeman N.C. (1991) Construction of three-dimensional stick figures from branched DNA. DNA Cell Biol. 10, 475–486 10.1089/dna.1991.10.475 1892564

[73] Rothemund P.W.K. (2006) Folding DNA to create nanoscale shapes and patterns. Nature 440, 297–302 10.1038/nature04586 16541064

[74] Saccà B., Meyer R., Erkelenz M., Kiko K., Arndt A., Schroeder H. et al. (2010) Orthogonal protein decoration of DNA origami. Angew. Chem. Int. Ed. Engl. 49, 9378–9383 10.1002/anie.201005931 21031395

[75] Nakata E., Liew F.F., Uwatoko C., Kiyonaka S., Mori Y., Katsuda Y. et al. (2012) Zinc-finger proteins for site-specific protein positioning on DNA-origami structures. Angew. Chem. Int. Ed. Engl. 2421–2424 10.1002/anie.201108199 22287266

[76] Dinh H., Nakata E., Lin P., Saimura M., Ashida H. and Morii T. (2019) Reaction of ribulose biphosphate carboxylase/oxygenase assembled on a DNA scaffold. Bioorganic Med. Chem. 27, 115120 10.1016/j.bmc.2019.115120 31627975

[77] Yoshidome T., Endo M., Kashiwazaki G., Hidaka K., Bando T. and Sugiyama H. (2012) Sequence-selective single-molecule alkylation with a pyrrole–imidazole polyamide visualized in a DNA nanoscaffold. J. Am. Chem. Soc. 134, 4654–4660 10.1021/ja209023u 22320236

[78] Tohgasaki T., Shitomi Y., Feng Y., Honna S., Emura T., Hidaka K. et al. (2019) A photocaged DNA nanocapsule for controlled unlocking and opening inside the cell. Bioconjug. Chem. 30, 1860–1863 10.1021/acs.bioconjchem.9b00040 30811178

[79] Ramakrishnan S., Ijäs H., Linko V. and Keller A. (2018) Structural stability of DNA origami nanostructures under application-specific conditions. Comput. Struct. Biotechnol. J. 16, 342–349 10.1016/j.csbj.2018.09.002 30305885 PMC6169152

[80] Setyawati M.I., Kutty R.V., Tay C.Y., Yuan X., Xie J. and Leong D.T. (2014) Novel theranostic DNA nanoscaffolds for the simultaneous detection and killing of *Escherichia coli* and *Staphylococcus aureus*. ACS Appl. Mater. Interfaces 6, 21822–21831 10.1021/am502591c 24941440

[81] Liu Y., Sun Y., Li S., Liu M., Qin X., Chen X. et al. (2020) Tetrahedral framework nucleic acids deliver antimicrobial peptides with improved effects and less susceptibility to bacterial degradation. Nano Lett. 20, 3602–3610 10.1021/acs.nanolett.0c00529 32272018

[82] Mela I., Vallejo-Ramirez P.P., Makarchuk S., Christie G., Bailey D., Henderson R.M. et al. (2020) DNA nanostructures for targeted antimicrobial delivery. Angew. Chem. Int. Ed. 59, 1269 10.1002/anie.202002740 PMC7496991 32297692

